# A convex formulation for joint RNA isoform detection and quantification from multiple RNA-seq samples

**DOI:** 10.1186/s12859-015-0695-9

**Published:** 2015-08-19

**Authors:** Elsa Bernard, Laurent Jacob, Julien Mairal, Eric Viara, Jean-Philippe Vert

**Affiliations:** 1 MINES ParisTech, PSL Research University, CBIO-Centre for Computational Biology, Fontainebleau, 77300 France; 2 Institut Curie, Paris, 75005 France; 3 INSERM U900, Paris, 75005 France; 4 Laboratoire Biométrie et Biologie Evolutive, Université de Lyon, Université Lyon 1, CNRS, INRA, UMR5558 Villeurbanne, France; 5 Inria, LEAR Team, Laboratoire Jean Kuntzmann, CNRS, Université Grenoble Alpes, 655, Avenue de l’Europe, Montbonnot, 38330 France; 6 Sysra, 91330 Yerres, France

**Keywords:** Isoform, RNA-seq, Alternative splicing, Muti-task estimation, Convex optimization

## Abstract

**Background:**

Detecting and quantifying isoforms from RNA-seq data is an important but challenging task. The problem is often ill-posed, particularly at low coverage. One promising direction is to exploit several samples simultaneously.

**Results:**

We propose a new method for solving the isoform deconvolution problem jointly across several samples. We formulate a convex optimization problem that allows to share information between samples and that we solve efficiently. We demonstrate the benefits of combining several samples on simulated and real data, and show that our approach outperforms pooling strategies and methods based on integer programming.

**Conclusion:**

Our convex formulation to jointly detect and quantify isoforms from RNA-seq data of multiple related samples is a computationally efficient approach to leverage the hypotheses that some isoforms are likely to be present in several samples. The software and source code are available at http://cbio.ensmp.fr/flipflop.

**Electronic supplementary material:**

The online version of this article (doi:10.1186/s12859-015-0695-9) contains supplementary material, which is available to authorized users.

## Background

Most genes in eukaryote genomes are subject to alternative splicing [1], meaning they can give rise to different mature mRNA molecules, called transcripts or isoforms, by including or excluding particular exons, retaining introns or using alternative donor or acceptor sites. Alternative splicing is a regulated process that not only greatly increases the repertoire of proteins that can be encoded by the genome [2], but also appears to be tissue-specific [3, 4] and regulated in development [5], as well as implicated in diseases such as cancers [6]. Hence, detecting isoforms in different cell types or samples is an important step to understand the regulatory programs of the cells or to identify splicing variants responsible for diseases.

Next-generation sequencing (NGS) technologies can be used to identify and quantify these isoforms, using the RNA-seq protocol [7–9]. However, identification and quantification of isoforms from RNA-seq data, sometimes referred to as the *isoform deconvolution problem*, is often challenging because RNA-seq technologies usually only sequence short portions of mRNA molecules, called *reads*. A given read sequenced by RNA-seq can therefore originate from different transcripts that share a particular portion containing the read, and a deconvolution step is needed to assign the read to a particular isoform or at least estimate globally which isoforms are present and in which quantity based on all sequenced reads.

When a reference genome is available, the RNA-seq reads can be aligned on it using a dedicated splice mapper [10–12], and the deconvolution problem for a given sample consists in estimating a small set of isoforms and their abundances that explain well the observed coverage of reads along the genome. One of the main difficulty lies in the fact that the number of candidate isoforms is very large, growing essentially exponentially with the number of exons. Approaches that try to perform *de novo* isoform reconstruction based on the read alignment include

Cufflinks [13], Scripture [14], IsoLasso [15], NSMAP [16], SLIDE [17], iReckon [18], Traph [19], MiTie [20], and FlipFlop [21]. However, the problem is far from being solved and is still challenging, due in particular to identifiability issues (the fact that different combinations of isoforms can correctly explain the observed reads), particularly at low coverage, which limits the statistical power of the inference methods: as a result, the performance reported by the state-of-the-art is often disappointingly low.

One promising direction to improve isoform deconvolution is to exploit several samples at the same time, such as biological replicates or time course experiments. If some isoforms are shared by several samples, potentially with different abundances, the identifiability issue may vanish and the statistical power of the deconvolution methods may increase due to the availability of more data for estimation. For example, the state-of-the-art methods CLIIQ [22] and MiTie [20] perform joint isoform deconvolution across multiple samples, by formulating the problem as an NP-hard combinatorial problem solved by mixed integer programming. MiTie avoids an explicit enumeration of candidate isoforms using a pruning strategy, which can drastically speed up the computation in some cases but remains very slow in other cases. The Cufflinks/Cuffmerge [13] method uses a more naive and straightforward approach, where transcripts are first predicted independently on each sample, before being merged (with some heuristics) in a unique set.

In this paper, we propose a new method for isoform deconvolution from multiple samples. When applied to a single sample, the method boils down to FlipFlop [21]; thus, we simply refer to the new multi-sample extension of the technique as FlipFlop as well. It formulates the isoform deconvolution problem as a continuous convex relaxation of the combinatorial problem solved by CLIIQ and MiTie, using the group-lasso penalty [23, 24] to impose shared sparsity of the models estimated on each sample. The group-lasso penalty allows to select a few isoforms among many candidates jointly across samples, while assigning sample-specific abundance values. By doing so, it shares information between samples but still considers each sample to be specific, without learning a unique model for all samples together as a merging strategy would do. Compared to CLIIQ or MiTie, FlipFlop addresses a convex optimization problem efficiently, and involves an automatic model selection procedure to balance the fit of the data against the number of detected isoforms. We show experimentally, on simulated and real data, that FlipFlop is more accurate than simple pooling strategies and than other existing methods for isoform deconvolution from multiple samples.

## Methods

The deconvolution problem for a single sample can be cast as a sparse regression problem of the observed reads against expressed isoforms, and solved by penalized regression techniques like the Lasso, where the *ℓ*_1_ penalty controls the number of expressed isoforms. This approach is implemented by several of the referenced methods, including IsoLasso [15] and FlipFlop [21]. When several samples are available, we propose to generalize this approach by using a convex penalty that leads to small sets of isoforms jointly expressed across samples, as we explain below.

### Multi-dimensional splicing graph

The splicing graph for a gene in a single sample is a directed acyclic graph with a one-to-one mapping between the set of possible isoforms of the gene and the set of paths in the graph. The nodes of the graph typically correspond to exons, sub-exons [15, 17, 20] or ordered sets of exons [21, 25]—the definition we adopt here as it allows to properly model long reads spanning more than 2 exons [21]. The directed edges correspond to links between possibly adjacent nodes.

**Fig. 1:**
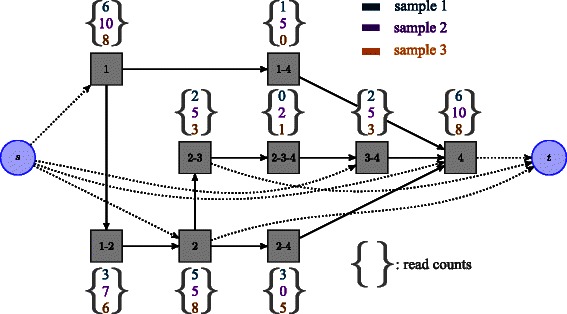
Multi-dimensional splicing graph with three samples. Each candidate isoform is a path from source node *s* to sink node *t*. Nodes denoted as grey squares correspond to ordered set of exons. Each read is assigned to a unique node, corresponding to the exact set of exons that it overlaps. Note that more than 2 exons can constitute a node, properly modeling reads spanning more than 2 exons. A vector of read counts (one component per sample) is then associated to each node of the graph. Note also that some components of a vector can be equal to zero

When working with several samples, we choose to build the graph based on the read alignments of all samples pooled together. Since the exons used to build the graph are estimated from read clusters, this step already takes advantage of information from multiple samples, and leads to a more accurate graph. We associate a list of read counts, as many as samples, with each node of the graph. In other words, we extend the notion of splicing graph to the multiple-sample framework, using a shared graph structure with specific count values on each node. Our multi-dimensional splicing graph is illustrated in Fig. 1.

### Notation

Throughout the paper, we call *G*=(*V*,*E*) the multi-dimensional splicing graph where *V* is the set of vertices and *E* the set of edges. We denote by $\mathcal {P}$ the set of all paths in *G*. By construction of the graph, each path $p \in \mathcal {P}$ corresponds to a unique candidate isoform. We denote by ${y_{v}^{t}}$ the number of reads falling in each node *v*∈*V* for each sample *t*∈{1,…,*T*}, where *T* is the number of samples. We denote by ${\beta _{p}^{t}} \in \mathbb {R}_{+}$ the abundance of isoform *p* for sample *t*. Finally, we define for every path *p* in $\mathcal {P}$ the *T*-dimensional vector of abundances $\boldsymbol \beta _{p} = \left [{\beta _{p}^{1}}, {\beta _{p}^{2}}, \ldots, {\beta _{p}^{T}}\right ]$, and denote by $\boldsymbol \beta = \left [\boldsymbol \beta _{p}\right ]_{p \in \mathcal {P}}$ the matrix of all abundances values with $|\mathcal {P}|$ rows and *T* columns.

### Joint sparse estimation

We propose to estimate ***β*** through the following penalized regression problem:

(1) $$ \min_{\boldsymbol \beta} ~~ \mathcal{L}(\boldsymbol \beta) + \lambda \sum_{p \in \mathcal{P}} \Arrowvert\, \boldsymbol \beta_{p} \,\Arrowvert{\!~\!}_{2} ~~~ \text{such that}~~ \boldsymbol \beta_{p} \geq 0 ~~\text{for all}\,{p} \in \mathcal{P},  $$

where $\mathcal {L}$ is a convex smooth loss function defined below, $\Arrowvert \, \boldsymbol \beta _{p} \,\Arrowvert _{2}=\sqrt {\sum _{t=1}^{T} \left ({\beta _{p}^{t}}\right)^{2}}$ is the Euclidean norm of the vector of abundances of isoform *p* across the samples, and *λ* is a non-negative regularization parameter that controls the trade-off between loss and sparsity. The *ℓ*_1,2_-norm $\|\boldsymbol \beta \|_{1, 2} = \sum _{p \in \mathcal {P}} \Arrowvert \, \boldsymbol \beta _{p} \,\Arrowvert _{2}$, sometimes called the group-lasso penalty [23], induces a shared sparsity pattern across samples: solutions of (1) typically have entire rows equal to zero [23], while the abundance values in the non-zero rows can be different among samples. This shared sparsity-inducing effect corresponds exactly to our assumption that only a limited number of isoforms are present across the samples (non-zero rows of *β*). It can be thought of as a convex relaxation of the number of isoforms present in at least one sample, which is used as criterion in the combinatorial formulations of CLIIQ and MiTie.

We define the loss function $\mathcal {L}$ as the sum of the *T* sample losses, thus assuming independence between samples as reads are sampled independently from each sample. The loss is derived from the Poisson negative likelihood (the Poisson model has been successfully used in several RNA-seq studies [16, 21, 26, 27]) so that the general loss is defined as

$$\mathcal{L}(\boldsymbol \beta)\,=\, \sum_{t=1}^{T} \sum_{v \in V} \left[{\delta_{v}^{t}}- {y_{v}^{t}} \log {\delta_{v}^{t}} \right] ~\text{with}~ {\delta_{v}^{t}} \,=\, \left(N^{t} l_{v}\! \sum_{p\in\mathcal{P} : p \ni v} \!\!\!{\beta_{p}^{t}}\right), $$

where *N* ^*t*^ is the total number of mapped reads in sample *t* and *l*_*v*_ is the effective length of node *v*, as defined in [21]. The sum $\sum {\beta _{p}^{t}}$ over all $p \in \mathcal {P}$ that contain node *v* represents the sum of expressions in sample *t* of all isoforms involving node *v*.

### Candidate isoforms

Since $|\mathcal {P}|$ grows exponentially with the number of nodes in *G*, we need to avoid an exhaustive enumeration of all candidate isoforms $p \in \mathcal {P}$. FlipFlop efficiently solves problem (1) in the case where *T*=1, *i.e.*, the *ℓ*_1_-regularized regression $ \min _{ \boldsymbol \beta _{p} \in \mathbb {R}_{+}} \mathcal {L}(\boldsymbol \beta) + \lambda \sum _{p \in \mathcal {P}} \beta _{p}$ using network flow techniques, without requiring an exhaustive path enumeration and leading to a polynomial-time algorithm in the number of nodes.

Unfortunately, this network flow formulation does not extend trivially to the multi-sample case. We therefore resort to a natural two-step heuristic: we first generate a large set of candidate isoforms by solving *T*+1 one-dimensional problems—the *T* independent ones, plus the one corresponding to all samples pooled together—for different values of *λ*, and taking the union of all selected isoforms, and we then solve (1) restricted to this union of isoforms. This approach can potentially miss isoforms which would be selected by solving (1) over all paths $p\in \mathcal {P}$ and are not selected for any single sample or when pooling all reads to form a single sample, but allows to efficiently approximate (1). We observe that it leads to good results in various settings in practice, as shown in the experimental part.

### Model selection

We solve (1) for a large range of values of the regularization parameter *λ*, obtaining solutions from very sparse to more dense (a sparse solution involves few non-zero abundance vectors ***β***_*p*_). Each solution, *i.e.*, each set of selected isoforms obtained with a particular *λ* value, is then re-fitted against individual samples—without regularization but keeping the non-negativity contraint—so that the estimated abundances do not suffer from shrinkage [28]. The solution with the largest BIC criterion [29], where the degree of freedom of a group-lasso solution is computed as explained in [23], is finally selected. Note that although the same list of isoforms selected by the group-lasso is tested on each sample, the refitting step lets each sample pick the subset of isoforms it needs among the list, meaning that all samples do not necessarily share *all* isoforms at the end of the deconvolution.

## Results and discussion

We show results on simulated human RNA-seq data with both increasing coverage and increasing number of samples, with different simulation settings, and on real RNA-seq data. In all cases, reads are mapped to the reference with TopHat2 [10]. We compare FlipFlop implementing the group-lasso approach (1) to the simpler strategy of pooling all samples together, running single-sample FlipFlop [21] on the merged data, and performing a fit for each individual sample data against the selected isoforms. We also assess the performance of MiTie [20] and of the version 2.2.0 of the Cufflinks/Cuffmerge package [13]. Performances on isoform identification are summarized in terms of Fscore, the harmonic mean of precision and recall, as used in other RNA-seq studies [20, 22]. Of note, in all the following experiments, we consider a *de novo* setting, without feeding any of the methods with prior transcript annotations (*i.e.*, MiTie and FlipFlop first reconstruct sub-exons and build the splicing graph, then perform isoform deconvolution).

### Influence of coverage and sample number

The first set of simulations is performed based on the 1329 multi-exon transcripts on the positive strand of chromosome 11 from the RefSeq annotation [30]. Single-end 150 bp reads are simulated with the RNASeqReadSimulator software (available at http://alumni.cs.ucr.edu/~liw/rnaseqreadsimulator.html). We vary the number of reads from 10 thousand – 10 million per sample (corresponding approximately to sequencing depth from 1 to 1000 ×) and the number of samples from 1 – 10. All methods are run with default parameters, except that we fix *region-filter* to 40 and *max-num-trans* to 10 in MiTie as we notice that choosing these two parameter values greatly increases its performances (see Additional file 1: Figure A.1 for a comparison between MiTie with default parameters or not).

**Fig. 2:**
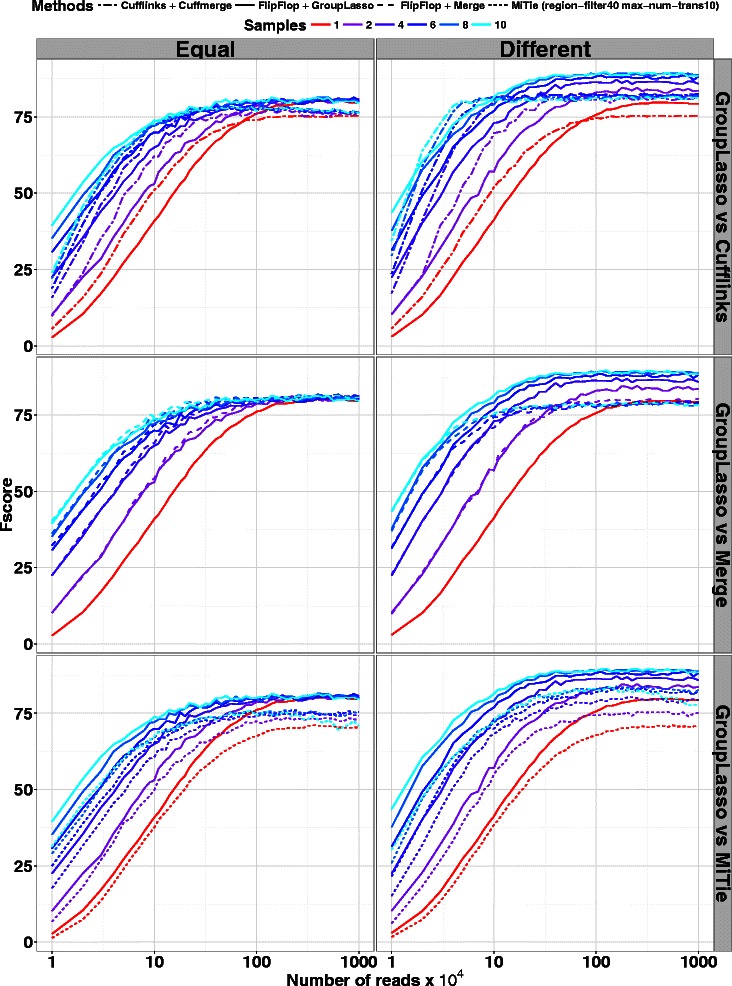
Human simulations with increasing coverage and number of samples

Figure 2 shows the Fscore in two different settings: the *Equal* setting corresponds to a case where all samples express the same set of transcripts at the same abundances (in other words each sample is a noisy realization of a unique abundance profile), while in the *Different* setting the abundance profiles of each sample are generated independently. Hence in that case the samples share the same set of expressed transcripts but have very different expression values (the maximum correlation between two abundance vectors is 0.088).

In all cases and for all methods, the higher the coverage or the number of samples, the higher the Fscore. In the *Equal* case, the group-lasso and merging strategies give almost identical results, which shows the good behavior of the group-lasso, as pooling samples in that case corresponds to learning the shared abundance profile. In the *Equal* case again, for all methods the different Fscore curves obtained with increasing number of samples converge to different plateaux. None of these levels reaches a Fscore of 100, but the group-lasso level is the highest (together with the merging strategy). In the *Different* case, the group-lasso shows equal or higher Fscore than the merging strategy, with a great improvement when the coverage or the number of samples increases. The group-lasso also outperforms the Cufflinks/Cuffmerge method for all numbers of samples when the coverage is larger than 80. When using more than 5 samples the group-lasso shows greater Fscore as soon as the coverage is bigger than 15 (see table B.1 of the supplementary material for statistical significance). Finally, the group-lasso outperforms MiTie for all number of samples and all coverages. Of note, the group-lasso performances are better in the *Different* setting than in the *Equal* setting, showing that our multi-sample method can efficiently deal with diversity among samples.

**Fig. 3:**
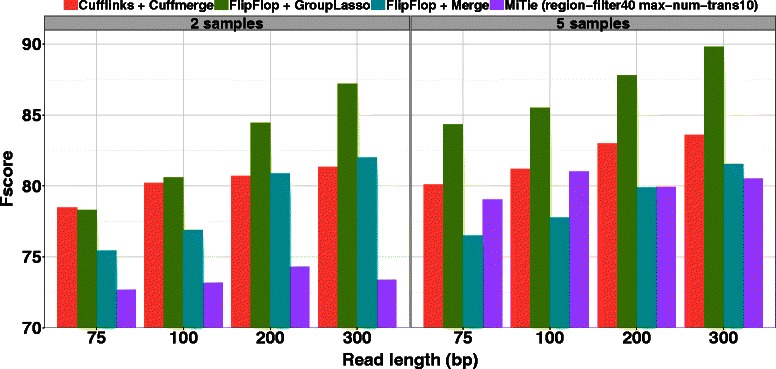
Human simulations with various read lengths

We also investigate the influence of the read length on the performance of the compared methods in the *Different* setting. Figure 3 shows the obtained Fscore when using either 2 or 5 samples with a fixed 100×10^4^ coverage, while read length varies from 75 to 300 bp. Because we properly model long reads in our splicing graph the group-lasso performance greatly increases with the read length, proportionally much more than other state-of-the-art methods. When using 5 samples and long 300 bp reads, the group-lasso reaches a very high Fscore of 90 (compared to 84 for the second best Cufflinks/Cuffmerge method), showing that our method is very well adapted to RNA-Seq design with long reads and several biological replicates.

Note finally that our method generalizes to paired-end reads. We show in Additional file 1: Figure C.1 a comparison of the tested methods on simulations in the *Different* setting using both paired or single-end reads at comparable coverage.

### Influence of hyper-parameters with realistic simulations

The second set of simulations is performed using a different and more realistic simulator, the Flux Simulator [31], in order to check that our approach performs well regardless the choice of the simulator. Coverage and single-end read length are respectively fixed to 10^5^ reads and 150 bp, and we run experiments for one up to five samples. We study the influence of hyper-parameters on the performances of the compared methods, and show that our approach leads to better results with optimized parameters as well. Hyper-parameters are first tuned on a training set of 600 transcripts from the positive strand of chromosome 11, which is subsequently left aside from the evaluation procedure after tuning. We start by jointly optimizing a set of pre-processing hyperparameters. We then keep the combination that leads to the best training Fscore, and we jointly optimize a set of prediction hyperparameters. More specifically, we optimize 7 values of 3 different pre-processing or prediction parameters (hence 7^3^ different combinations in both cases), except that for MiTie we add 2 values of one pre-processing parameter and 3 values of a fourth prediction parameter (hence optimizing over 9×7^2^ and 3×7^3^ parameters). A more detailed description of the optimized parameters is given in tables D.1 and D.2 of the supplementary.

**Fig. 4:**
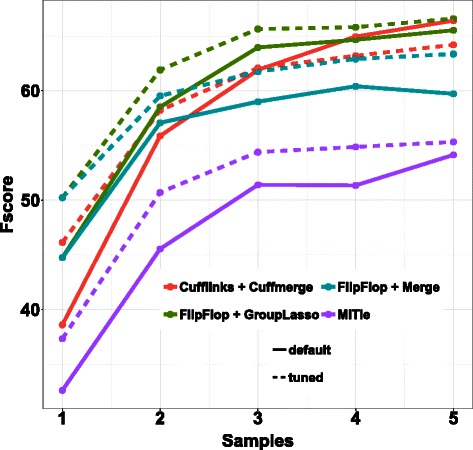
Fscore results on the Flux Simulator simulations

Fscore is shown on Fig. 4 for 600 other test transcripts, for both default and tuned settings (except that again we set *region-filter* to 40 and *max-num-trans* to 10 in MiTie instead of using all default parameters as it greatly improves its performances, see Additional file 1: Figure A.2 for a comparison of several versions of MiTie). For all methods and for both default and tuned settings, performances increase with the number of samples. Except for Cufflinks/Cuffmerge for the last three sample numbers, all methods improve their results after tuning of their hyper-parameters. When using default parameter values, the group-lasso shows the largest Fscore for the first three sample numbers, while Cufflinks/Cuffmerge is slightly better for the very last sample number. When using tuned parameter values, the group-lasso approach outperforms all other methods for the first three sample numbers, and is slightly better or equal to the default version of Cufflinks/Cuffmerge for the last two sample numbers.

### Experiments with real data

We use five samples from time course experiments on *D. melanogaster* embryonic development. Each sample corresponds to a 2-hour period, from 0–10 h (0–2 h, 2–4 h, …, 8–10 h). Data is available from the modENCODE [32] website. For each given period we pooled all 75 bp single-end technical replicate reads available, ending up with approximately 25 – 45 million mapped reads per sample. A description of the samples is given in table C.1. Data from the same source were also used in the MiTie paper [20].

Because the exact true sets of expressed transcripts is not known, we validated predictions based on public transcript annotations. We built a comprehensive reference using three different databases available on the UCSC genome browser [33], namely the RefSeq [30], Ensembl [34] and FlyBase [35] annotations. More specifically, we took the union of the multi-exon transcripts described in the three databases, while considering transcripts with the same internal exon/intron structure but with different length of the first or the last exon as duplicates. Reads were mapped to the reference transcriptome in order to restrict predictions to known genomic regions, and we perform independent analysis on the forward and reverse strands. All methods are run with default parameters.

**Fig. 5:**
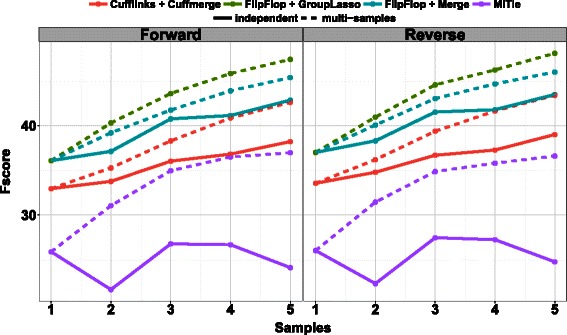
Fscore results on the modENCODE data

Figure 5 shows the Fscore per sample when FlipFlop, MiTie, and Cufflinks are run independently on each sample or when multi-sample strategies are used. Results on the forward and reverse strands are extremely similar. All methods give better results than their independent versions, and the performances of the multi-sample approaches increase with the number of used samples. Again, the group-lasso strategy of FlipFlop seems more powerful than the pooling strategy, and gives better Fscore than MiTie and Cufflinks/Cuffmerge in that context.

Considering running time, each method was run on a 48 CPU machine at 2.2 GHz with 256 GB of RAM using 6 threads (all tools support multi-threading). When using only a single sample and 6 threads, Cufflinks, FlipFlop and MiTie respectively completed in ∼4.2 min, ∼9.5 min and ∼26.6 min. while when using 5 samples and 6 threads, Cufflinks/Cuffmerge, FlipFlop with group-lasso and MiTie took ∼0.45 h, ∼1 h and ∼25 h(see Additional file 1: Figure G.1).

### Illustrative examples

**Fig. 6:**
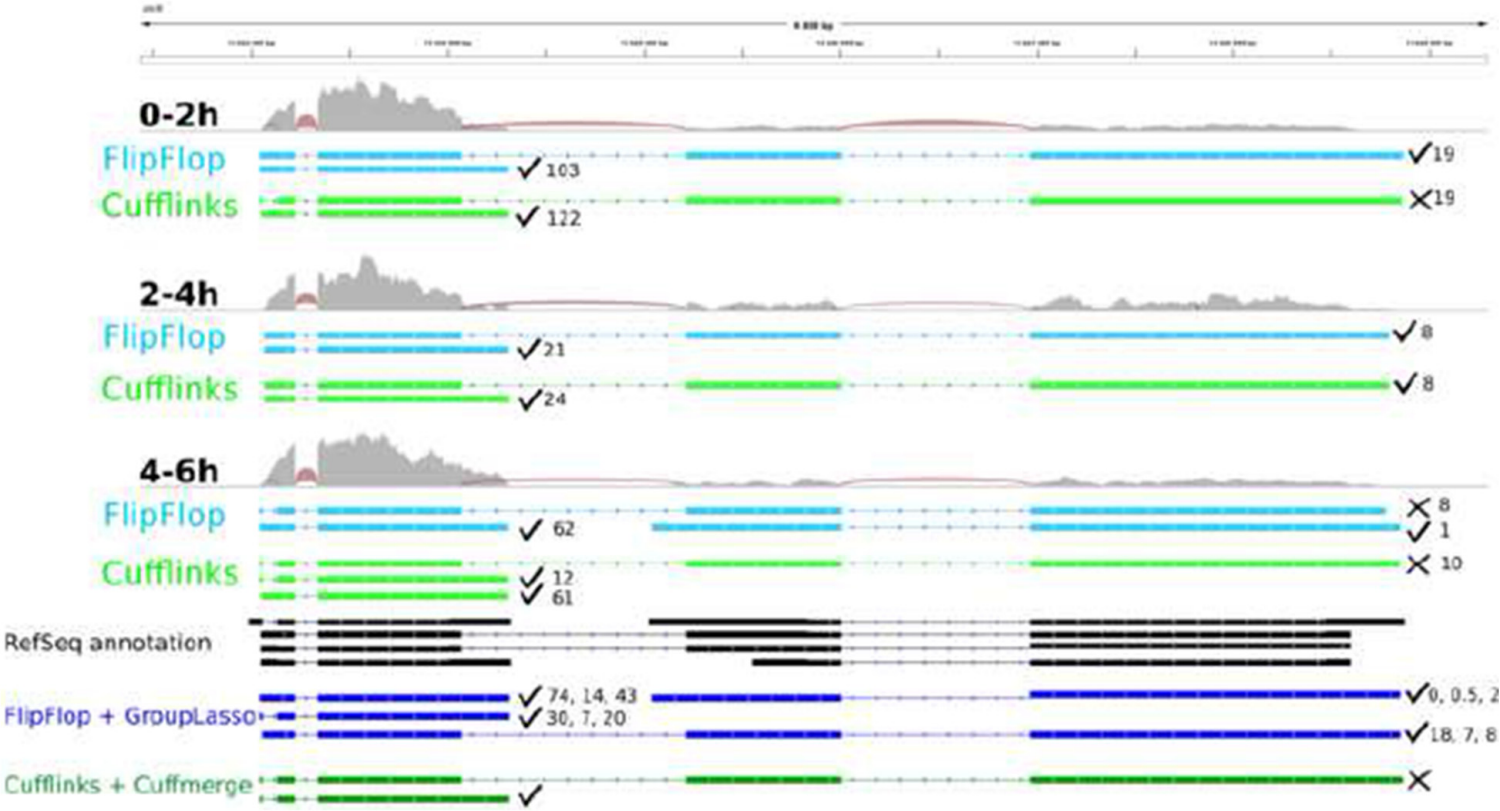
Transcriptome predictions of gene CG15717 from 3 samples of the modENCODE data. Samples name are 0–2 h, 2–4 h and 4–6 h. Each sample track contains the read coverage (light grey) and junction reads (red) as well as FlipFlop predictions (light blue) and Cufflinks predictions (light green). The bottom of the figure displays the RefSeq records (black) and the multi-sample predictions of the group-lasso (dark blue) and of Cufflinks/Cuffmerge (dark green)

We describe an example as a proof of concept that multi-sample FlipFlop with the group-lasso approach (1) can be much more powerful in some cases than its independent FlipFlop version, and than the merging strategy of Cufflinks/Cuffmerge. Figure 6 shows transcriptome assemblies of gene CG15717 on the first three modENCODE samples presented in the previous section, denoted as 0–2 h, 2–4 h and 4–6 h on the figure. For each sample, we display the read coverage along the gene, the junctions between exons, and the single-sample FlipFlop and Cufflinks predictions. At the bottom of the figure, we show the 6 RefSeq records as well as the multi-sample predictions obtained with FlipFlop or with Cuffmerge. A predicted transcript is considered as valid if all its exon/intron boundaries match a RefSeq record (✓ and ✗ denote validity or not). The estimated abundances in FPKM are given on the right-hand side of each predicted transcript. Of note, the group-lasso predictions come with estimated abundances (one specific value per sample), whereas Cufflinks/Cuffmerge only reports the structure of the transcripts.

For single-sample predictions, FlipFlop and Cufflinks report the same number of transcripts for each sample (respectively 2, 2 and 3 predictions for samples 0–2 h, 2–4 h and 4–6 h), with the same number of valid transcripts, except for the first sample where FlipFlop makes 2 good guesses against 1 for Cufflinks. This difference might be due to the fact that FlipFlop not only tries to explain the read alignement as Cufflinks does, but also the coverage discrepancies along the gene.

For multi-sample predictions, FlipFlop gives much more reliable results, with 4 validated transcripts (among 4 predictions), while Cufflinks/Cuffmerge makes only 1 good guess out of 2 predictions. FlipFlop uses evidences from all samples together to find transcripts with for instance missing junction reads in one of the sample (such as the one with 30, 7 and 20 FPKM) or lowly expressed transcripts (such as the one with 0, 0.5 and 2 FPKM). Cufflinks/Cuffmerge explains all read junctions but does not seek to explain the multi-sample coverage, which seems important in that example.

Importantly, one can note that the results of multi-sample group-lasso FlipFlop are different from the union of all single-sample FlipFlop predictions (the union coincides here to the results of FlipFlop on the merged sample—data not shown). This illustrates the fact that designing a dedicated multi-sample procedure can lead to more statistical power than merging individual results obtained on each sample independently. We display an additional example in Additional file 1: Figure H.1.

## Conclusion

We proposed a multi-sample extension of FlipFlop, which implements a new convex optimization formulation for RNA isoform identification and quantification jointly across several samples. Experiments on simulated and real data show that an appropriate method for joint estimation is more powerful than a naive pooling of reads across samples. We also obtained promising results compared to MiTie, which tries to solve a combinatorial formulation of the problem.

Accurately estimating isoforms in multiple samples is an important preliminary step to differential expression studies at the level of isoforms [36, 37]. Indeed, isoform deconvolution from single samples suffers from high false positive and false negatives rates, making the comparison between different samples even more difficult if isoforms are estimated from each sample independently. Although the FlipFlop formulation of joint isoform deconvolution across samples provides a useful solution to define a list ofisoforms expressed (or not) in each sample, variants of FlipFlop specifically dedicated to the problem of finding differentially expressed isoforms may also be possible by changing the objective function optimized in (1).

Finally, as future multi-sample applications such as jointly analyzing large cohorts of cancer samples or many cells in single-cell RNA-seq are likely to involve hundreds or thousands of samples, more efficient implementations involving in particular distributed optimization may be needed.

## Additional file

Additional file 1
**This file provides additional results on the simulated experiments, as well as a detailed description of the real data, and more illustrative examples.** (PDF 1505 kb)
