# Supplementary material for: A convex formulation for joint RNA isoform detection and quantification from multiple RNA-seq samples
Source: BMC Bioinformatics. 2015 Aug 19;16:262. doi: 10.1186/s12859-015-0695-9 (PMC4543468; doi:10.1186/s12859-015-0695-9)
Supplement: Additional file 1 — This file provides additional results on the simulated experiments, as well as a detailed description of the real data, and more illustrative examples. (PDF 1505 kb) [file 12859_2015_695_MOESM1_ESM.pdf]

Supplementary material for “A convex formulation for joint RNA isoform detection and quantification from multiple RNA-seq samples”

Elsa Bernard[1,2,3], Laurent Jacob[4], Julien Mairal[5], Eric Viara[6], Jean-Philippe Vert[1,2,3]

June 29, 2015

Appendices

A Some influence of MiTie parameters on human simultations

B Statistical significance on human simulations

C Paired-end simulations

D Parameter optimization on human simulations

E Description of real RNA-seq data

F Abundance comparison on real RNA-seq data

G Running time on real RNA-seq data

H More illustrative examples

[1]Centre for Computational Biology – CBIO, Mines ParisTech, Fontainebleau, France, [2]Institut Curie, Paris, France, [3]INSERM U900, Paris, France, [4]LBBE, Lyon, France, [5]LEAR Project-Team, INRIA Grenoble - Rhône Alpes, France, [6]Sysra, Yerres, France

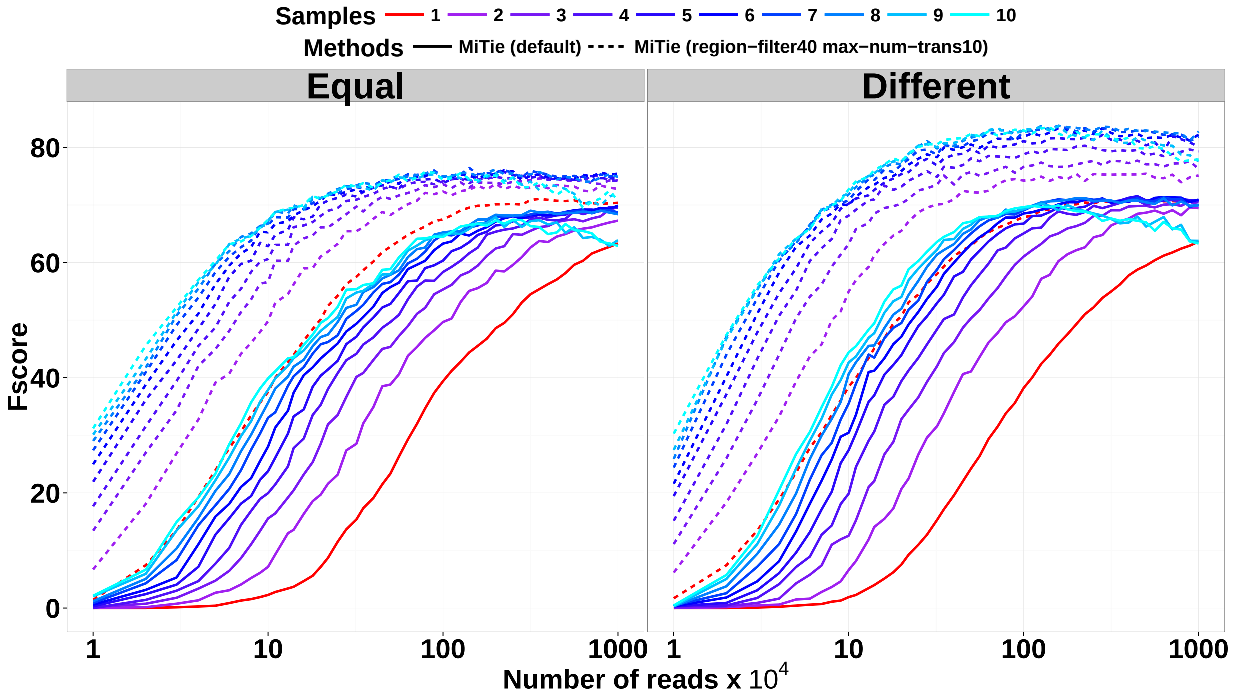

Figure A.1: MiTie results on a first set of human simulations when using default parameters or setting *region-filter* to 40 and *max-num-trans* to 10.

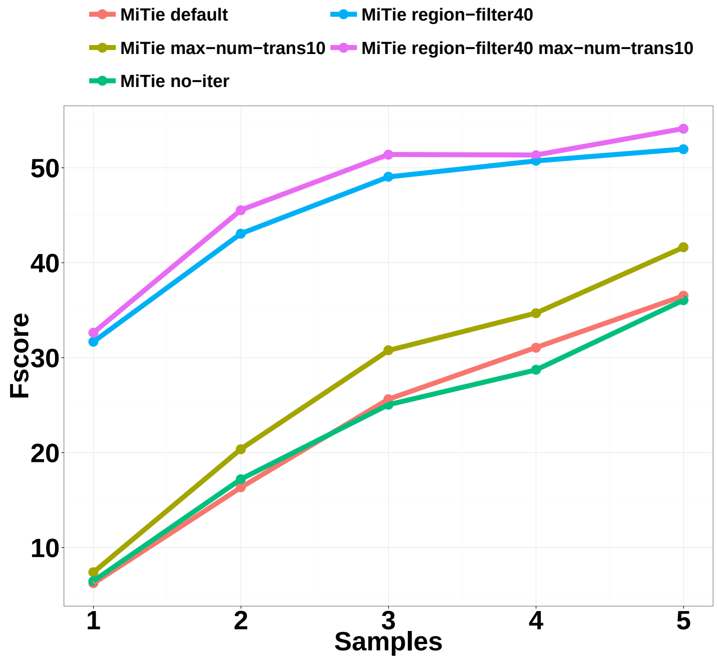

Figure A.2: MiTie results on a second set of human simulations when varying some parameters.

	1-3 samples	4-6 samples	7-10 samples
1-10 coverage ($\times 10^4$ reads)	1	1	1
10-50 coverage ($\times 10^4$ reads)	1	0.035	$< 10^{-16}$
50-100 coverage ($\times 10^4$ reads)	0.040	$< 10^{-16}$	$< 10^{-16}$
100-1000 coverage ($\times 10^4$ reads)	$< 10^{-16}$	$< 10^{-16}$	$< 10^{-16}$

Table B.1: Statistical testing to assess performances in the *Different* human simulation setting, for different ranges of coverage and number of samples. Numbers correspond to the BH adjusted p-values when testing the null hypothesis that the Fscore obtained with FlipFlop+GroupLasso are lower than the Fscore obtained with Cufflinks+Cuffmerge (one-sided paired t-test). Note that when testing FlipFlop+GroupLasso against MiTie, all adjusted p-values are extremely small.

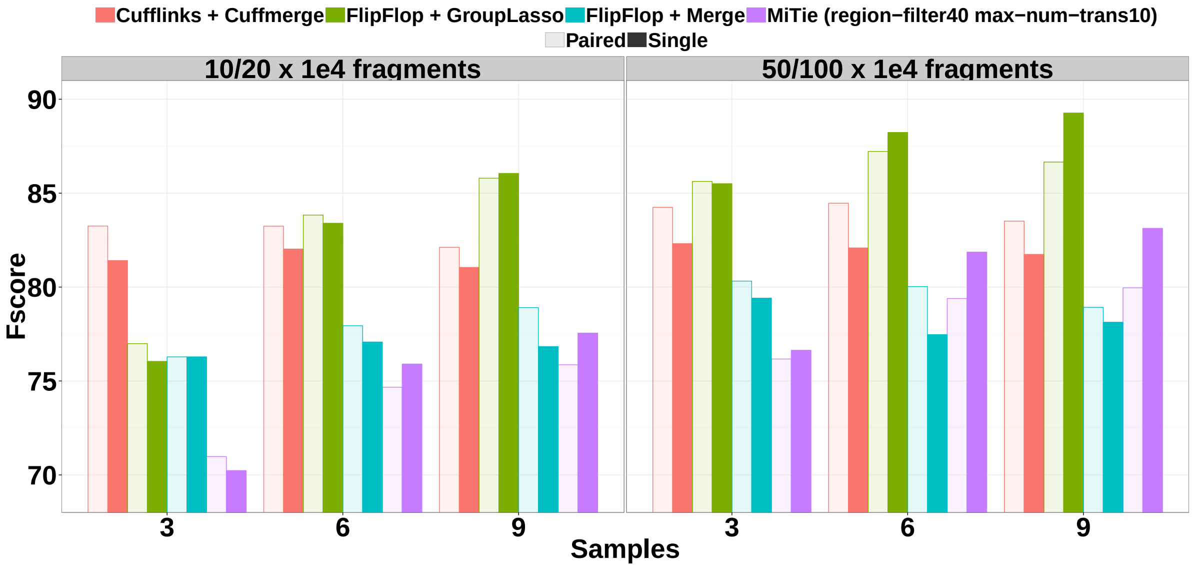

Figure C.1: Simulation using both paired or single-end reads at comparable coverage. The legend 10/20 or 50/100 represents $10^4\times$ the number of sequenced fragments in the paired-end setting versus the single-end setting (the number of sequenced reads is then equal in the two settings, while the number of sequenced fragments is twice higher is the single-end setting). The read length is fixed to 150bp and the mean fragment size to 350bp in the paired-end setting.

Overall, our FlipFlop+GroupLasso method clearly achieves the best performances in both the paired and single-end settings in the high 50/100 coverage case, and also in the low 10/20 coverage case when using 9 samples.
For Cufflinks+Cuffmerge, the paired-end setting is systematically a bit better than the single-end one, while for both FlipFlop+GroupLasso and MiTie the two settings are either comparable or better in the single-end case.

Methods	Pre-processing parameters (with default values)	Optimal values for each number of samples				
		1	2	3	4	5
MiTie	region-filter (1000)	50	50	50	50	10
	seg-filter (0.05)	0.01	0.01	0.01	0.01	0.01
	tss-tts-pval (10^{-4})	6×10^{-5}	6×10^{-5}	2×10^{-5}	6×10^{-5}	6×10^{-5}
Cufflinks	min-frags-per-transfrag (10)	29	17	17	17	29
	max-multiread-fraction (0.75)	0.15	0.15	0.15	0.15	0.15
	overlap-radius (50)	146	85	85	85	146
FlipFlop + Merge	minReadNum (40)	23	40	23	8	14
	minJuncCount (1)	1	1	1	1	1
	minCvgCut (0.05)	0.02	0.03	0.01	0.01	0.01
FlipFlop + GroupLasso	minReadNum (40)	23	40	23	8	14
	minJuncCount (1)	1	1	1	1	1
	minCvgCut (0.05)	0.02	0.01	0.01	0.01	0.01

Table D.1: Details on the optimized pre-processing parameters.

Methods	Prediction parameters (with default values)	Optimal values for each number of samples				
		1	2	3	4	5
MiTie	max-num-trans (2)	5	5	10	10	10
	C-exon (10)	29	50	17	50	29
	C-intron (100)	100	20	58	292	171
	C-num-trans (100)	20	20	20	20	34
Cufflinks	min-isoform-fraction (0.10)	0.02	0.03	0.02	0.02	0.02
	pre-mrna-fraction (0.15)	0.08	0.08	0.03	0.03	0.03
	junc-alpha (10^{-3})	2×10^{-4}	2×10^{-4}	2×10^{-4}	2×10^{-4}	2×10^{-4}
FlipFlop + Merge	BICcst (50)	10	50	50	85	50
	cutoff (1)	0	1	1	3	3
	delta (10^{-7})	10^{-11}	10^{-11}	10^{-10}	10^{-10}	10^{-11}
FlipFlop + GroupLasso	BICcst (50)	10	29	29	50	50
	cutoff (1)	0	0	0	0	1
	delta (10^{-7})	10^{-11}	10^{-9}	10^{-10}	10^{-10}	10^{-10}

Table D.2: Details on the optimized prediction parameters.

Sample descriptions	SRA accession names	Total number of reads mapped on the reference transcriptome
0-2h embryos	SRR023659<br>SRR023755<br>SRR023671<br>SRR023663<br>SRR023747	25 388 344
2-4h embryos	SRR023722<br>SRR023745<br>SRR023705<br>SRR023660	24 541 397
4-6h embryos	SRR023746<br>SRR023836<br>SRR023696<br>SRR023669<br>SRR035220	46 722 946
6-8h embryos	SRR023691<br>SRR023732<br>SRR023654<br>SRR023668<br>SRR024217	32 231 644
8-10h embryos	SRR023754<br>SRR023657<br>SRR023749<br>SRR023701<br>SRR023759<br>SRR024219<br>SRR023750	29 544 727

Table E.1: Description of the *D.melanogaster* RNA-seq data from the modENCODE project. Data can be found at the following adress: `http://intermine.modencode.org/query/experiment.do?experiment=Developmental+Time+Course+Transcriptional+Profiling+of+D.+melanogaster+Using+Illumina+poly\%28A\%29\%2B+RNA-Seq`

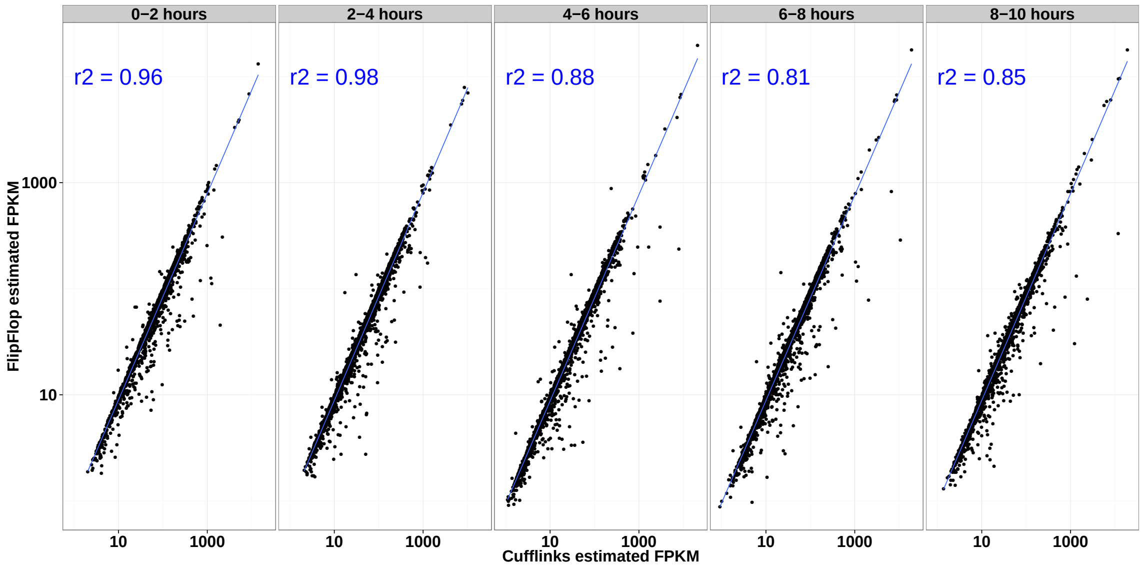

Figure F.1: Abundance estimation on the five samples of *D.melanogaster* RNA-seq data (forward strand). Scatter-plot are between the one-sample FlipFlop method and Cufflinks for genes where both method found the same set of expressed transcripts.

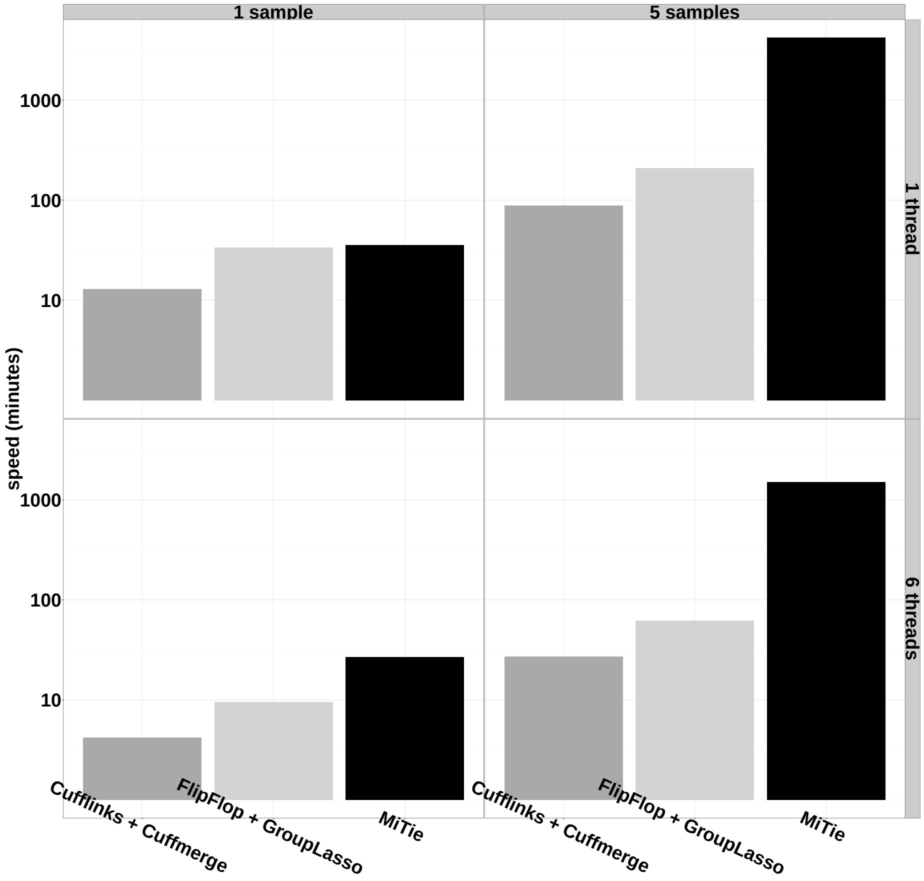

Figure G.1: Running time on the *D.melanogaster* RNA-seq data (forward strand). Each method was run on a 48 CPU machine at 2.2GHz with 256GB of RAM, on either 1 or 6 threads (all tools support multi-threading). MiTie is more than 20 times slower than FlipFlop+GroupLasso when using 5 samples.

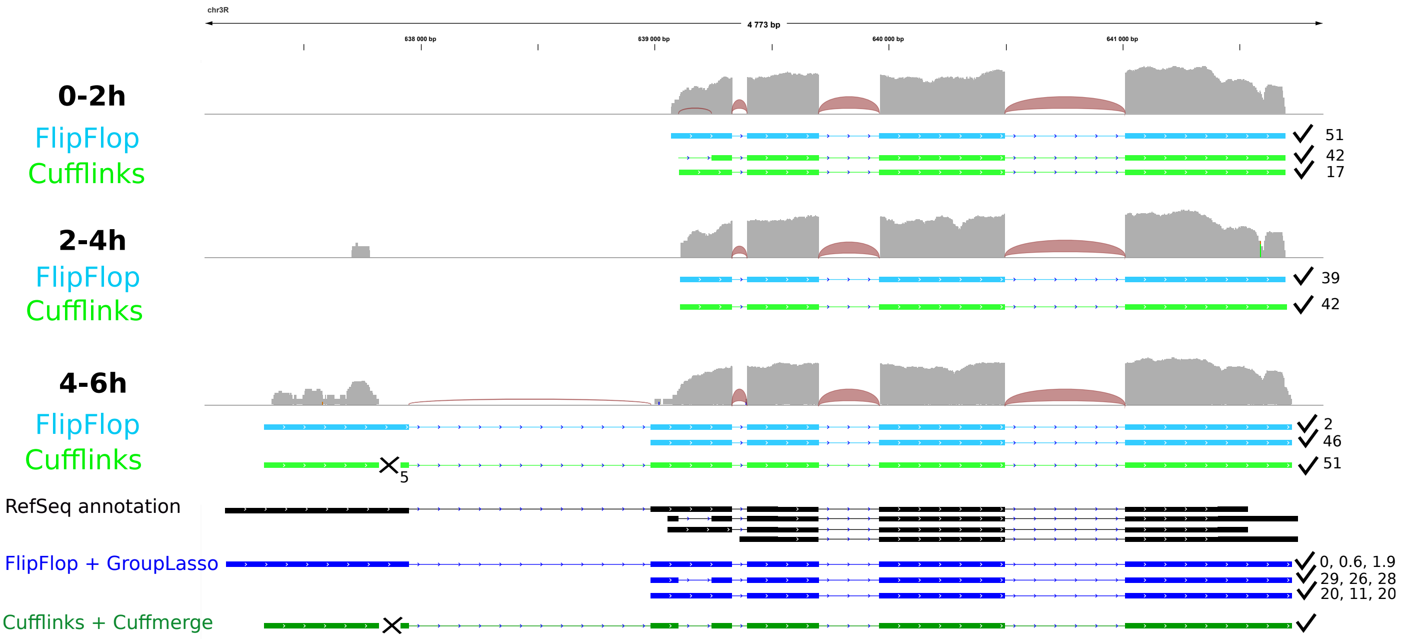

Figure H.1: Transcriptome predictions of gene CG1129 from 3 samples of the modENCODE data. Samples name are 0-2h, 2-4h and 4-6h. Each sample track contains the read coverage (light grey) and junction reads (red) as well as FlipFlop predictions (light blue) and Cufflinks predictions (light green). Here coverage is log-scale. The bottom of the figure displays the RefSeq records (black) and the multi-sample predictions of the group-lasso (dark blue) and of Cufflinks/Cuffmerge (dark green). Symbols ✓ and ✗ indicate if a predicted transcript matches a RefSeq record of not. Estimated abundances in FPKM are given on the right hand side of each transcript.

Figure H.1 illustrates that our group-lasso approach can be more powerful than indivual predictions and than the merging strategy of Cuffmerge. Indeed, when using evidences from several samples (both junctions and coverage discrepancies) our approach finds a lowly expressed transcript (that was found in only 1 sample with individual predictions), and two well expressed transcripts, including one that was not previously found with individual predictions. On the other hand, Cufflinks/Cuffmerge is very conservative and only predicts a long transcript that does not explain the variations of coverage from the left to the right part of the gene.
